# Orchestrating the metastatic symphony: the role of extracellular vesicles in the epithelial–mesenchymal transition and pulmonary niche formation of breast cancer

**DOI:** 10.1002/brv.70167

**Published:** 2026-04-02

**Authors:** Jian Lu, Reza Shirazi Nia, Junjie Sun, Chrysa Filippopoulou, Daniel De Vega, Pengtao Hu, Heng Liang, Hang Yao, Chaoyue Pan, Xiaoyan Wu, Xidong Gu, Xiaohong Xie, Georgios Giamas

**Affiliations:** ^1^ International Oncology Institute, The First Affiliated Hospital of Zhejiang Chinese Medical University, Oncology Department of the First Affiliated Hospital of Zhejiang Chinese Medical University Hangzhou 310053 China; ^2^ Department of General Surgery Nanjing First Hospital, Nanjing Medical University Nanjing 210006 China; ^3^ Department of Breast Surgery The First Affiliated Hospital of Zhejiang Chinese Medical University Hangzhou 310053 China; ^4^ Department of Biochemistry and Biomedicine School of Life Sciences, University of Sussex Brighton UK

**Keywords:** breast cancer, lung metastasis, epithelial–mesenchymal transition, extracellular vesicles, exosomes, pre‐metastatic niche, cell–cell communication, organotropism, molecular sorting

## Abstract

The complexity of breast cancer (BC) lung metastasis lies in the capacity of tumour cells to interact efficiently with distant organs to promote colonisation, a process that involves the sophisticated coordination of inherent cellular plasticity and the remodelling of the distant microenvironment. This review emphasises the essential function of extracellular vesicles (EVs) within this communication network. Tumour‐derived EVs (TEVs) not only induce epithelial–mesenchymal transition (EMT) by reprogramming breast cancer cell gene expression networks, thereby enhancing migratory and invasive capabilities, but also serve as a ‘vanguard’, arriving in the lungs in advance to educate stromal cells and establish a pre‐metastatic niche that facilitates breast cancer progression. This review uniquely conceptualises EV‐mediated EMT and niche formation as a synergistic and sequential biological continuum. We comprehensively examine the sorting mechanisms of EV molecular cargo, targeted delivery approaches, and hierarchical regulatory networks. Critically, we propose that the concurrent regulation of EMT and niche formation is likely driven by the synergistic action of distinct EV subpopulations rather than single ‘multitasking’ vesicles. Future investigations dissecting this heterogeneity will be pivotal for verifying this synergistic subpopulations hypothesis and establishing a theoretical basis for precise EV‐based metastasis intervention strategies.

## I. INTRODUCTION

The multi‐step cellular process of breast cancer (BC) metastasis includes crucial phases such as local invasion, intravasation, circulatory survival, extravasation, and distant implantation. For cancer cells to complete successfully their metastatic journey from the source location to distant organs, each stage depends on accurate intercellular communication networks (Naxerova, 2025; Rabas *et al*., 2024; Entenberg, Oktay & Condeelis, 2023; Gerstberger, Jiang & Ganesh, 2023). Among these, the unique organotropism of BCs towards the lungs is a critical biological phenomenon that remains only partially understood. It involves intricate intercellular connections and microenvironmental remodelling (McGinnis *et al*., 2024; Houthuijzen & de Visser, 2022; Xiao *et al*., 2025; Hoshino *et al*., 2015). The epithelial–mesenchymal transition (EMT), a crucial step of cellular plasticity in BC metastasis, and confers upon cancer cells the ability to migrate, invade, and acquire stem cell‐like properties, which initiates the metastatic cascade (Kumar *et al*., 2024; Kang *et al*., 2025; Khanicheragh *et al*., 2024; Liu *et al*., 2021a; Li *et al*., 2022; Li *et al*., 2025; Xie *et al*., 2023; Raymond *et al*., 2025). Subsequently, EMT‐generated migratory cancer cells intravasate into the circulatory system. In the meantime, by altering the pulmonary milieu, tumour‐derived extracellular vesicles (TEVs) encourage metastatic implantation (Shang, Zhang & Chao, 2025).

Extracellular vesicles (EVs), as highly efficient mediators of intercellular communication, precisely orchestrate the BC metastasis cascade by transporting diverse biomolecules – including proteins, nucleic acids, lipids, and metabolites – thereby emerging as fundamental regulators of the metastatic process (Shang *et al*., 2025; Lee *et al*., 2023; Xu *et al*., 2024; Simon, Jackson & Giamas, 2020a). In addition to maintaining physiological intercellular communication, EVs are ‘hijacked’ as tumours grow, changing from a physiological mechanism to a core engine driving pathological processes. Recent studies demonstrate that EVs accurately influence downstream target gene networks and core EMT transcription factors like zinc finger E‐box binding homeobox 1/2 (ZEB1/2), SNAIL, SNAIL family transcriptional repressor 2 (SLUG) and TWIST through multimodal synergistic effects (Kang *et al*., 2025; Liu *et al*., 2021a; Li *et al*., 2022). In the EMT regulatory network, this integrated delivery system transforms EVs from passive carriers to active coordinators, providing a fresh viewpoint on the intercellular communication processes that underlie BC metastasis (Shang *et al*., 2025).

This review will examine the integration of EVs and their role in two pivotal processes: EMT and the development of the pre‐metastatic lung microenvironment, establishing a comprehensive regulatory network across molecular, cellular, and tissue levels. Through systematic mapping of EV‐mediated intercellular communication pathways, we aim to elucidate key molecular regulatory elements involved in BC metastasis. Specifically, we will elucidate how EVs intricately regulate EMT progression and modify the pulmonary microenvironment through multivariate synergistic interactions involving proteins, nucleic acids, lipids, and metabolites. This approach provides new insights into the biological nature of metastasis and establishes the theoretical foundation for the development of EV‐based intervention strategies to combat metastasis.

## II. EV‐MEDIATED REPROGRAMMING OF CANCER CELLS: ORCHESTRATING THE EMT PROGRAMME

We categorise these processes according to the cellular origin of the vesicles (tumour‐derived *versus* stroma‐derived) and the specifics of their molecular payloads (proteins *versus* nucleic acids) in order to clarify the intricacy of this reprogramming.

### (1) Biogenesis and heterogeneity: the definition of the term ‘EVs’

EVs are a diverse class of naturally occurring particles that are encased in lipid bilayers and released by almost every type of cell. EVs can be roughly divided into two major types, exosomes and ectosomes (also known as microvesicles), based on their size and biogenesis mechanisms. Exosomes (see Table 1 for glossary), which have a diameter of 30–150 nm, originate from the endosomal system. Intraluminal vesicles (ILVs) are formed by membrane invagination of multivesicular bodies (MVBs) during their biogenesis. This process is strictly controlled by the endosomal sorting complex required for transport (ESCRT), its related proteins, such as programmed cell death 6‐interacting protein (ALIX) and tumour susceptibility 101 (TSG101), and tetramembrane proteins like cluster of differentiation 63 (CD63), cluster of differentiation 81 (CD81), and cluster of differentiation 9 (CD9). After the MVB and plasma membrane fuse, these ILVs are discharged as exosomes into the extracellular environment (Shang *et al*., 2025; Welsh *et al*., 2024).

**Table 1:** Glossary of core concepts in EV‐mediated breast cancer lung metastasis.

Ectosomes (microvesicles)	A subtype of extracellular vesicles (EVs) (typically 100 nm–1 μm) formed by the direct outward budding and fission of the plasma membrane.
Exosomes	Small EVs (typically 30–150 nm) originating from the endosomal system, specifically formed *via* the inward budding of the limiting membrane of multivesicular bodies (MVBs).
Organotropism	The biological phenomenon where metastatic tumour cells exhibit a specific affinity for colonising particular distant organs (e.g. breast cancer preferentially metastasising to the lungs, bone, or brain).
Pre‐metastatic niche (PMN)	A supportive microenvironment induced in distant organs by the primary tumour (*via* secreted factors or EVs) prior to the arrival of tumour cells, facilitating their subsequent survival and colonisation.
The ‘postal code’ hypothesis	The concept that specific surface proteins on EVs (such as integrins) act as molecular ‘postal codes’, directing the vesicles to specific organs or tissues.
Vascular remodelling	The process of altering the structure and permeability of blood vessels (e.g. in the lung) to facilitate tumour cell extravasation, often induced by EV‐delivered proteases and angiogenic factors.

By contrast, ectosomes or microvesicles (generally 100 nm to 1 μm) are formed *via* the direct outward budding and fission of the plasma membrane, thereby enclosing cytosolic material in the process (Willms *et al*., 2018). Although historical literature often employs the term ‘exosomes’ to denote all secreted vesicles, current guidelines [Minimal Information for Studies of Extracellular Vesicles (MISEV2023); Welsh *et al*., 2024] recommend the use of operational terminology based on size (e.g. small EVs, <200 nm) or biochemical composition, as the physical properties of these subgroups tend to overlap (Welsh *et al*., 2024). In this review, we employ the collective term ‘EVs’ to refer to these vesicular populations, reserving the term ‘exosomes’ solely for references to studies that explicitly isolated this subpopulation using established markers.

### (2) EV cargo: a molecular toolkit for inducing cellular plasticity

#### (a) Protein payloads: hijacking canonical EMT pathways

**Fig. 1:**
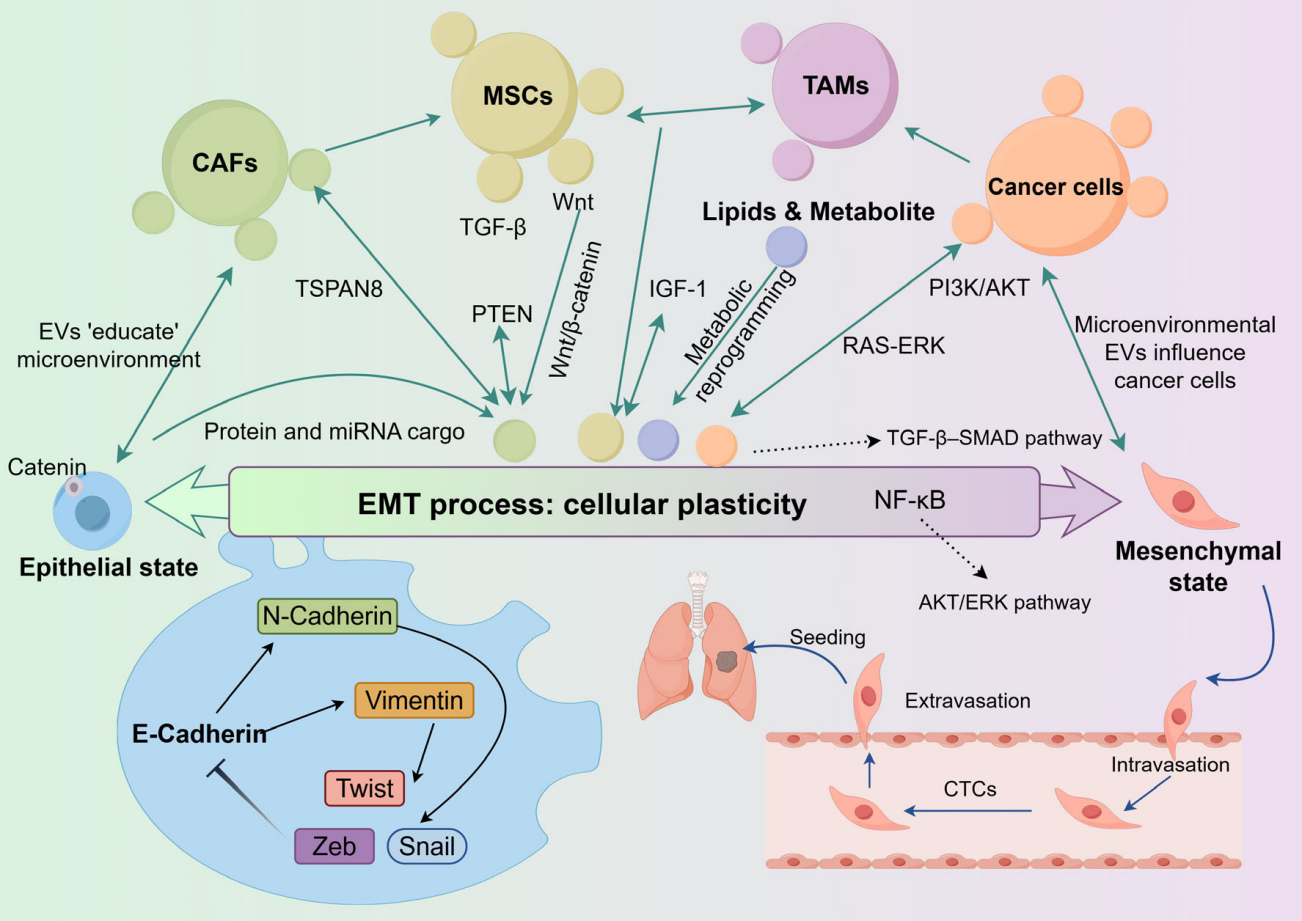
Illustration of the regulation of the epithelial–mesenchymal transition in breast cancer by extracellular vesicle‐mediated networks of intercellular communication. During the transition, the central gradient arrow displays the transition from polygonal epithelial cells with high E‐cadherin expression (left) to spindle‐shaped mesenchymal cells (right) with high N‐cadherin expression. The bidirectional communication networks are established by extracellular vesicles that originate from cancer cells, cancer‐associated fibroblasts, mesenchymal stem cells, and tumour‐associated macrophages, as illustrated in the schematic. The signalling pathways and transcription factors that are involved in the downregulation of E‐cadherin and the upregulation of vimentin are illustrated in the figure. In summary, the pulmonary microenvironment is influenced by extracellular vesicles, while migratory cancer cells enter the circulatory system through intravascular and extravascular escape in the bottom right panel. Abbreviations: AKT, protein kinase B; CAFs, cancer‐associated fibroblasts; CTCs, circulating tumour cells; EMT, epithelial–mesenchymal transition; ERK, extracellular signal‐regulated kinase; EVs, extracellular vesicles; IGF‐1, insulin‐like growth factor 1; miRNA, microRNA; MSC, mesenchymal stem cell; NF‐κB, nuclear factor kappa B; PI3K, phosphoinositide 3‐kinase; PTEN, phosphatase and tensin homolog; RAS, rat sarcoma virus; SMAD, small mothers against decapentaplegic; TAMs, tumour‐associated macrophages; TGF‐β, transforming growth factor β; TSPAN8, tetraspanin 8; Zeb, zinc finger E‐box binding homeobox.

BC cell‐derived EVs initiate and maintain the EMT process by co‐opting canonical EMT signalling pathways through the transfer of essential signalling proteins (Fig. 1). Research suggests that EVs derived from BC cells contain proteins associated with the transforming growth factor β (TGF‐β) signalling pathway, which induce CD8+ T‐cell exhaustion through TGF‐β receptor II‐mediated signalling and indirectly promote EMT within the tumour microenvironment (Xie *et al*., 2022). Additionally, EVs have the ability to transfer important Wingless‐Int (Wnt) signalling pathway proteins, which activates the Wnt/β‐catenin axis and subsequently promotes EMT (Ma *et al*., 2024). Webber *et al*. (2010) found that TEVs can promote EMT within the tumour microenvironment by inducing fibroblast development into myofibroblasts, a process that shares molecular pathways with EMT (Webber *et al*., 2010). In BC, EV‐mediated activation of signalling pathways – particularly TGF‐β and Wnt pathways – triggers the expression of crucial transcription factors such as SNAIL, ZEB1/2, and TWIST, thereby initiating the EMT programme (Becker *et al*., 2016). Parvanian *et al*. (2025) demonstrated that TEVs carry the mesenchymal protein vimentin to promote EMT. As a signature EMT marker, the presence of vimentin demonstrates that EVs can directly transport EMT‐associated proteins, helping to promote the development of a mesenchymal phenotype (Parvanian *et al*., 2025).

In addition to these soluble components, transmembrane proteins on the surface of TEVs are also important. By controlling the E‐cadherin/catenin signalling pathway in recipient cells, the membrane protein tetraspanin 8 (TSPAN8), which is abundant in TEVs and highly expressed in BC cells, facilitates metastasis. This suggests that the surface proteins and luminal contents of TEVs work together to trigger EMT (Voglstaetter *et al*., 2019). However, a crucial question regarding the functional importance of this transport system remains. Since EV‐shuttled signalling molecules, like TGF‐β and Wnt, are also widely distributed in soluble form within the tumour microenvironment, it is unclear whether EV‐encapsulated signalling confers distinct biological advantages, such as protection against degradation or enabling high‐concentration local delivery, or whether it is merely a redundant pathway. Identifying the precise roles of vesicular transport in EMT induction will require further research that focuses on differentiating these effects.

#### (b) The non‐coding RNA regulome: a symphony of post‐transcriptional control

EVs contribute to the initiation and maintenance of EMT by means of specific post‐transcriptional regulatory networks, as they are carriers of non‐coding RNAs. By controlling E‐cadherin expression, Myelocytomatosis viral oncogene homolog/ N‐myc avian myelocytomatosis viral oncogene neuroblastoma derived homolog (MYC/MYCN)‐activated microRNA (miRNA) miR‐9 stimulates EMT and metastasis in BC cells (Ma *et al*., 2010). In BC, EVs can cause recipient cells to undergo EMT by transferring miR‐9 from tumour cells to recipient cells. On the other hand, the EMT process can be inhibited by the miR‐200 family, which can be transferred intercellularly through EVs (Samuels *et al*., 2023). By stimulating the Wnt and nuclear factor kappa B2 (NF‐κB2) pathways, long non‐coding RNAs (lncRNAs) like AL139294.1 can be transported to recipient cells *via* EVs and encourage cancer cells to behave malignantly (Ma *et al*., 2024). Through the ‘miRNA sponge’, circular RNAs (circRNAs) are included as an EV cargo and contribute to the formation of intricate EMT regulatory networks by binding particular miRNAs and modifying the expression of target genes (Samuels *et al*., 2023).

#### (c) Lipids and metabolites: emerging conductors of EMT

Little is known about the function of lipids and metabolites carried by EVs in EMT. According to current research, some lipids, such as 25‐hydroxycholesterol, may promote EMT by affecting cellular metabolic reprogramming. According to Dou *et al*. (2025), the transfer of lipids and metabolites may be a key factor in EV‐mediated intercellular communication that controls tumour fate within the tumour microenvironment. Furthermore, the metabolic states of recipient cells can be changed by EV‐mediated metabolite transfer, encouraging the production of phenotypes linked to EMT (Dou *et al*., 2025). However, the exact processes by which certain lipids transported by EVs control EMT are still unknown. Technological developments, especially in single‐EV analysis methods (Saftics *et al*., 2023; Lin *et al*., 2025; Nikoloff, Saucedo‐Espinosa & Dittrich, 2023; Rima *et al*., 2022), will enable more precise characterisation of how lipids and metabolites within EVs modulate EMT in the future. It is noteworthy that the majority of studies, including those concerning lipids, employed bulk EV isolation techniques. Consequently, it remains uncertain whether an EMT‐inducing capability is an inherent characteristic of all secreted vesicles or if it relies on an uncommon but potent subpopulation. This deficiency in single‐vesicle resolution presently constrains our capacity to assign particular metabolic reprogramming functions to individual EV subpopulations.

### (3) Dialogues in the tumour microenvironment: feedback and amplification of EMT

The metastatic cascade in BC is primarily driven by intercellular communication within the tumour microenvironment. EVs are essential molecular carriers that use complex feedback loops to control EMT accurately. EVs that come from stromal cells, including tumour‐associated macrophages (TAMs) and cancer‐associated fibroblasts (CAFs), contain a particular molecular payload that includes lipids, proteins, and miRNAs. This cargo creates a long‐lasting positive feedback loop by altering the gene expression profiles of BC cells and triggering and intensifying the EMT process (Liu *et al*., 2023a; Eguchi *et al*., 2022; Zhang *et al*., 2024; Lin *et al*., 2024b).

Through the delivery of various chemical payloads, cell‐derived EVs serve a crucial regulatory function in the EMT process of BC. In terms of nucleic acid payloads, research shows that by targeting transcription elongation factor A like 7 (*TCEAL7*), CAF‐derived exosomal miR‐18b dramatically increases the invasive and metastatic potential of BC cells. This mechanism is especially noticeable in triple‐negative breast cancer (TNBC) (Yan *et al*., 2021). These stromal‐derived EVs transport Wnt signalling ligands to activate the β‐catenin pathway, which in turn promotes the production of transcription factors linked to EMT and enhances cancer cell motility (Liu *et al*., 2023a).

Concerning BC subtype specificity, TEVs of various molecular subtypes demonstrate significant differences in their capacity to induce EMT. Exosomes derived from low‐metastatic subclones can ‘educate’ highly metastatic subclones by activating the Wnt signalling pathway to facilitate pulmonary metastasis, a phenomenon confirmed in the 4 T1 mouse BC model (Li *et al*., 2022). Similarly, by targeting receptor‐like tyrosine kinase (*RYK*) and participating in the non‐canonical Wnt pathway, downregulation of exosomal miR‐7‐5p encourages migration and invasion in BC cells. This mechanism has been reported in human epidermal growth factor receptor 2 (HER2)‐positive BC (Liang *et al*., 2022). Notably, mRNAs encoding EMT‐associated transcription factors such as SNAIL, TWIST, and ZEB are frequently found in higher concentrations in cell‐derived EVs from TNBC and HER2‐positive BC, which is in accordance with their clinical features of earlier visceral metastasis (Liu *et al*., 2021a; Luo *et al*., 2023).

Moreover, tumour heterogeneity is essential for the regulation of EV‐mediated EMT. EV‐mediated molecular exchange amongst BC cell subpopulations is known to result in the emergence of dynamic signalling networks that promote adaptive cancer cell evolution. For example, downregulated circPOKE enhances EMT progression and metastatic capacity during cancer evolution by activating the ubiquitin‐specific protease 10 (USP10)–SNAIL axis (Luo *et al*., 2023). Simultaneously, TSPAN8 is strongly expressed in small EVs produced from epithelial cancer cells, which facilitates improved uptake and restricted diffusion, hence increasing molecular interchange between different subtypes of tumour cells (Wang *et al*., 2021).

These results demonstrate that EMT can be precisely regulated by EV‐mediated molecular communication within the tumour microenvironment, creating a multi‐layered feedback amplification network. Through their special cargo‐sorting processes, EVs not only act as communication bridges between stromal and cancer cells, but also send microenvironmental cues to tumour cells, starting and maintaining EMT. In addition to explaining the organ tropism of BC lung metastases, this dynamic molecular exchange network provides fresh perspectives on the genesis of tumour heterogeneity and suggests possible treatment approaches that target EV‐mediated EMT pathways (Shang *et al*., 2025; Steinbichler *et al*., 2019; Samuels *et al*., 2022). Collectively, these findings highlight that TEVs do not merely reflect the donor cell's content but selectively package specific oncogenic cargoes effectively to hijack the EMT programme in recipient cells.

## III. THE ‘PRE‐METASTATIC OVERTURE’: HOW EVs ENGINEER THE PULMONARY NICHE

### (1) The ‘postal code’: molecular basis of EV homing to the lung

The organ‐specific tropism in tumour metastasis continues to represent a fundamental challenge in our understanding of the progression of cancer. EVs operate as a ‘postal code’ system, employing surface molecules to direct their precise delivery to particular organ microenvironments (Cheytan *et al*., 2025; Urabe *et al*., 2021; Nadeau *et al*., 2025). Pioneering research (Hoshino *et al*., 2015) established integrins as the primary ‘postal code’ responsible for directing organ‐specific EV localisation. In BC lung metastasis, integrins α6β1 and α6β4 function as the primary address codes on EVs, specifically guiding their interaction with the laminin‐rich pulmonary microenvironment (Hoshino *et al*., 2015).

In particular, laminin‐rich pulmonary microenvironments are the preferred target of BC exosomes containing integrin α6β1, which promotes fibroblast activation and extracellular matrix remodelling (Hoshino *et al*., 2015). Additionally, integrin α6β4 expression can be upregulated by small BC cell‐derived EVs enriched in caveolin‐1 (CAV1), which improves EV uptake by pulmonary epithelial cells (Lin *et al*., 2024a). These findings underscore the essential role of integrin subtype specificity in organ tropism. Other integrin subunits such as αvβ5 are associated with hepatic chemotaxis, thereby emphasising the precision of integrin ‘coding’ in determining organ preference (Wang *et al*., 2024a).

Beyond integrins, CD44 – a principal surface molecule on BC‐derived exosomes – has been identified as a vital mediator of lung‐specific homing. CD44 specifically interacts with hyaluronic acid, which is prevalent in lung tissue, thereby promoting the retention and internalisation of exosomes within pulmonary fibroblasts, endothelial cells, and immune cells (Kesharwani *et al*., 2021; Johnson *et al*., 2018). This selective adhesion not only improves the efficiency of EV uptake but also facilitates immunosuppression and angiogenesis within the pulmonary microenvironment (Moloudizargari, Asghari & Goel, 2021). Although direct evidence for EV‐associated CD44 in BC lung metastasis remains limited, tumour cell expression of CD44 is associated with increased metastatic potential, underscoring its general significance in lung‐targeted colonisation (Magoling *et al*., 2023).

Although the ‘postal code’ theory mediated by integrins (e.g. α6β4) offers a compelling molecular mechanism for chemotaxis, it is essential to distinguish this active targeting from passive accumulation. Due to the haemodynamic characteristics of pulmonary capillaries, the lung functions as the principal site for the non‐specific physical retention of circulating particles. Therefore, future investigations into chemotaxis must meticulously control for haemodynamic parameters to ensure that EV retention is genuinely attributable to specific receptor–ligand interactions rather than vascular filtration.

### (2) Educating the lung stroma: preparing the ‘soil’ for the metastatic ‘seed’

#### (a) Vascular remodelling: EVs increase pulmonary vascular permeability by carrying pro‐angiogenic factors and proteases

**Fig. 2:**
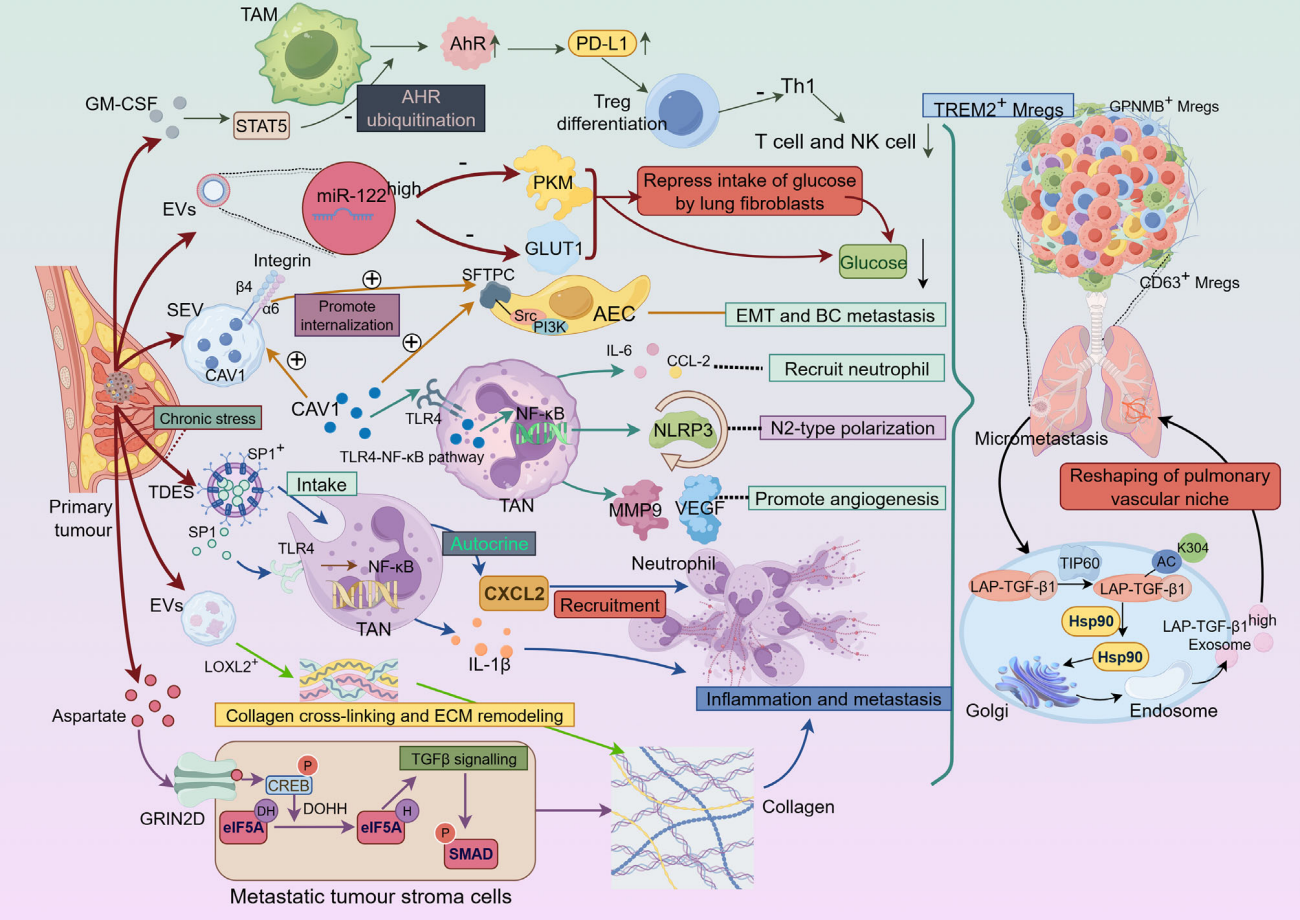
The cellular and molecular interactions within the pulmonary microenvironment during breast cancer lung metastasis. These interactions are mediated by extracellular vesicles. Primary tumour cells secrete a variety of extracellular vesicles and other factors, including aspartate and GM‐CSF, that interact with a variety of pulmonary stromal and immune cells. These cells include tumour‐associated macrophages, tumour‐associated neutrophils, alveolar epithelial cells, and fibroblasts. The figure illustrates a variety of signalling axes, such as the miR‐122‐mediated effect on glucose metabolism, integrin and CAV1‐mediated internalisation, the TLR4–NF‐κB pathway in neutrophils, CREB‐dependent DOHH expression that results in collagen deposition, and the transport of LAP–TGF‐β1 from the Golgi apparatus to endosomes. The micrometastasis and reshaping of the pulmonary vascular niche, which involve a variety of cellular populations, are depicted in the right panel. Abbreviations: AC, acetylation; AEC, alveolar epithelial cell; AhR, aryl hydrocarbon receptor; BC, breast cancer; CAV1, caveolin‐1; CCL2, C‐C motif chemokine ligand 2; CREB, cAMP response element‐binding protein; CXCL2, C‐X‐C motif chemokine ligand 2; DOHH, deoxyhypusine hydroxylase; eIF5A, eukaryotic initiation factor 5A; EVs, extracellular vesicles; GLUT1, glucose transporter 1; GM‐CSF, granulocyte‐macrophage colony‐stimulating factor; GPNMB, glycoprotein NMB; GRIN2D, glutamate ionotropic receptor NMDA type subunit 2D; Hsp90, heat shock protein 90; IL‐1β, interleukin‐1 beta; IL‐6, interleukin‐6; LAP, latency associated peptide; miR, microRNA; MMP9, matrix metalloproteinase 9; Mregs, regulatory macrophages; NF‐κB, nuclear factor kappa B; NK cell, natural killer cell; NLRP3, NOD‐like receptor family pyrin domain containing 3; PD‐L1, programmed death‐ligand 1; PI3K, phosphoinositide 3‐kinase; PKM, pyruvate kinase; SEV, small extracellular vesicle; SFTPC, surfactant protein C; SMAD, small mothers against decapentaplegic; SP1, specificity protein 1; Src, proto‐oncogene tyrosine‐protein kinase Src; STAT5, signal transducer and activator of transcription 5; TAM, tumour‐associated macrophage; TAN, tumour‐associated neutrophil; TDES, tumour‐derived exosomes; TGF‐β, transforming growth factor β; Th1, T helper 1 cell; Tip60, Tat‐interactive protein 60 kDa; TLR4, Toll‐like receptor 4; Treg, regulatory T cell; TREM2, triggering receptor expressed on myeloid cells 2; VEGF, vascular endothelial growth factor.

As essential mediators of intercellular communication, TEVs govern pulmonary vascular remodelling through the transportation of various molecular constituents (Fig. 2). EVs facilitate angiogenesis and increase vascular permeability by delivering protein cargoes [such as vascular endothelial growth factor (VEGF) and matrix metalloproteinases (MMPs)] and regulatory miRNAs, thereby establishing conducive conditions for tumour cell metastatic colonisation (Khanicheragh *et al*., 2024). Through their miRNA and protein cargo, TEVs reprogram recipient cells, increasing tissue permeability, thereby promoting cancer cell‐specific organotropism. ADP‐ribosylation factor 1 (ARF1) overexpression (connected to MMP‐9 activation), phosphorylation of ras homolog family member A (RhoA) and ras homolog family member C (RhoC) GTPases, and extracellular matrix metalloproteinase inducer (EMMPRIN)‐dependent p38 phosphorylation are key molecular mechanisms driving this permeability (Kang *et al*., 2025). Lung metastasis is further supported by genes like cyclooxygenase‐2 (*COX‐2*), *MMP‐1*, and *MMP‐2*, which stimulate angiogenesis and disrupt pulmonary capillary integrity (Bos *et al*., 2009; Minn *et al*., 2007).

#### (b) Matrix remodelling: EVs activate pulmonary fibroblasts to induce extracellular matrix component secretion

TEVs are instrumental in the formation of pre‐metastatic compartments in the lung, modifying the physical characteristics of pulmonary tissue through the induction of matrix remodelling (Kong *et al*., 2019; Liu *et al*., 2023b; Keklikoglou *et al*., 2019). Tumour cell metastasis and implantation are facilitated by factors transported by EVs, such as pro‐angiogenic proteins (MMPs, VEGF) and regulatory miRNAs, which not only promote angiogenesis but also take part in matrix remodelling processes (Khanicheragh *et al*., 2024). In BC lung metastasis, lysyl oxidase like 2 (LOXL2)‐mediated pre‐metastatic niche formation represents a crucial mechanism of extracellular matrix remodelling (Salvador *et al*., 2017). These TEVs modify the pulmonary microenvironment *via* their molecular payload, facilitating the deposition and reorganisation of extracellular matrix components to establish an appropriate ‘soil’ for metastatic tumour cells (Fig. 2).

## IV. INTEGRATIVE PERSPECTIVE: THE TANDEM EFFECT OF EMT AND NICHE FORMATION

### (1) Coordinated process: do EMT‐inducing EVs also prime the niche?

EMT and the creation of the pre‐metastatic niche are two important interconnected steps in the complex process of BC metastasis (Ryu *et al*., 2024; Jiang *et al*., 2024; McGinnis *et al*., 2024). As critical mediators facilitating communication between tumour cells and their surrounding microenvironment, do EVs concurrently orchestrate the induction of EMT and the preparation of the pre‐metastatic niche? The fundamental question is whether EMT‐inducing EVs and their descendant EVs demonstrate disparities in their cargo composition and functional properties. Do these vesicles represent a single population of multitasking ‘messengers’, or distinct subpopulations engaging in specialised collaboration? To facilitate a more comprehensive understanding of these molecular coordinators, Table 2 delineates the most extensively characterised EV cargoes, their cellular sources, and verified downstream targets.

**Table 2:** Key extracellular vesicle (EV) shuttling molecules regulating epithelial–mesenchymal transition (EMT) and pre‐metastatic microenvironment formation in breast cancer (BC).

Cargo type	Molecule	Cellular origin	Primary functional effects	Downstream targets/pathways	References
Proteins	Transforming growth factor β (TGF‐β)/Wingless‐Int (Wnt) ligands	Breast cancer (BC) cells	Induction of epithelial–mesenchymal transition (EMT); T cell exhaustion	Small mothers against decapentaplegic (SMAD) 2/3 pathway; Wnt/β‐catenin axis	Xie *et al*. (2022); Ma *et al*. (2024)
	Vimentin	BC cells	Promotion of mesenchymal phenotype	Cytoskeletal remodelling (direct delivery)	Parvanian *et al*. (2025)
	Tetraspanin 8 (TSPAN8)	BC cells	Promotion of metastasis	E‐cadherin/catenin signalling	Voglstaetter *et al*. (2019)
	Integrin α6β1/α6β4	BC cells	Lung organotropism; niche formation	Laminin binding; fibroblast activation	Hoshino *et al*. (2015)
	Caveolin‐1 (CAV1)	BC cells	Niche formation; neutrophil recruitment	Toll‐like receptor 4 (TLR4)–nuclear factor kappa B (NF‐κB)–interleukin‐6 (IL‐6)/C‐C motif chemokine ligand 2 (CCL2) axis	Lin *et al*. (2024a)
	Ephrin type‐A receptor 2 (EPHA2)	High‐metastatic BC cells	Angiogenesis	Ephrin A1–EPHA2; AMP‐activated protein kinase (AMPK) pathway	Han *et al*. (2022)
	Specificity protein 1 (SP1) (transcription Factor)	Tumour cells (under chronic stress)	Immunosuppression (neutrophil polarisation)	TLR4–NF‐κB pathway; IL‐1β secretion	Zhang *et al*. (2025)
MicroRNAs (miRNAs)	miR‐9	BC cells	Induction of EMT	E‐cadherin	Ma *et al*. (2010)
	miR‐18b	Cancer‐associated fibroblasts (CAFs)	Promotion of invasion and metastasis	Transcription elongation factor A like 7 (TCEAL7) (downregulation)	Yan *et al*. (2021)
	miR‐122	BC cells	Metabolic reprogramming of niche	Pyruvate kinase (PKM); Glucose transporter 1 (GLUT1)	Fong *et al*. (2015)
	miR‐106a‐5p	Triple‐negative BC (TNBC) cells	Selective enhancement of EMT	Target genes associated with EMT	Ansieau, (2013)
	miR‐451a	BC cells	Lung fibrosis; niche conditioning	Reprogramming of lung epithelial cells	Jeong *et al*. (2022)
	miR‐7‐5p	BC cells (low levels)	Migration and invasion (Human epidermal growth factor receptor 2 [HER2] + BC)	Receptor‐like tyrosine kinase (RYK); non‐canonical Wnt pathway	Liang *et al*. (2022)
Long non‐coding RNAs (lncRNAs)	AL139294.1	BC cells	Oncogenic behaviour promotion	Wnt and NF‐κB2 pathways	Ma *et al*. (2024)

Recent studies suggest that EVs may simultaneously fulfill both functions. In terms of EMT regulation, TEVs contain a variety of molecular components that might directly cause epithelial cells to undergo EMT, such as certain RNAs, proteins, and lipids. For example, EVs derived from MDA‐MB‐231 cell lines treated with linoleic acid caused MCF10A cells to adopt an EMT‐like phenotype (Galindo‐Hernandez *et al*., 2014); miR‐18b‐enriched EVs facilitate EMT by activating NF‐κB to regulate SNAIL protein; CAF‐derived EVs abundant in miR‐181d‐5p promote EMT through targeting *CDX2* and suppressing *HOXA5* (Yan *et al*., 2021; Wang *et al*., 2020); EVs overexpressing TSPAN8 promote cell migration and EMT by increasing adhesion efficiency to target cells (Johnson *et al*., 2018; Moloudizargari *et al*., 2021; Magoling *et al*., 2023; Shen & TanTai, 2024); when exosomes overexpress TGF‐β2, both benign and malignant breast epithelial cells undergo EMT (Qin *et al*., 2016).

Through a variety of processes, TEVs alter the pulmonary microenvironment in terms of niche development. First, pulmonary fibroblasts, endothelial cells, and other cells internalise EVs, which causes them to become activated and secrete different substances that modify the extracellular matrix (Kochetkova & Samuel, 2022; Chen *et al*., 2021). Second, TEVs can increase vascular permeability and trigger pulmonary angiogenesis by carrying pro‐angiogenic factors and proteases (Adnani *et al*., 2022; Han *et al*., 2022; Duan *et al*., 2021). Thirdly, by delivering immunoregulatory molecules (such as programmed death‐ligand 1, PD‐L1), TEVs create an immunosuppressive environment in the lung, functioning as a sanctuary for cancer cells (Li *et al*., 2021; Yang *et al*., 2018; Qiu *et al*., 2021; Li *et al*., 2024).

EV‐mediated EMT and niche development are not totally independent. In addition to increasing lung epithelial cell absorption by upregulating integrin α6β4 expression, EVs produced from BC cells, especially those enriched in CAV1, also attract neutrophils by activating the Toll‐like receptor 4 (TLR4)–NF‐κB–interleukin‐6 (IL‐6)/C‐C motif chemokine ligand 2 (CCL2) axis (Fig. 2). This causes type 2 polarisation mediated by NOD‐like receptor family pyrin domain containing 3 (NLRP3) and simultaneously promotes angiogenesis *via* MMP‐9 and VEGF‐A (Lin *et al*., 2024a). Moreover, the glucose uptake of pre‐metastatic lung stromal cells is inhibited by miR‐122‐rich EVs (Fong *et al*., 2015). Aspartic acid released by tumours triggers cAMP response element‐binding protein (CREB)‐dependent deoxyhypusine hydroxylase (DOHH) expression, which increases TGF‐β–small mothers against decapentaplegic (SMAD) pathway activity through eukaryotic initiation factor 5A (eIF5A) regulation and causes collagen deposition in micrometastatic foci (McGinnis *et al*., 2024; Doglioni *et al*., 2025); Latency associated peptide (LAP)‐TGF‐β1 in EVs derived from lung metastases undergoes tat‐interactive protein 60 kDa (Tip60)‐mediated acetylation at position K304, interacting with heat shock protein 90α (Hsp90α) to promote its transport from the Golgi apparatus to endosomes and enrichment within exosomes, ultimately remodelling the pulmonary vascular microenvironment (Yu *et al*., 2024) (Fig. 2).

These results imply that EVs might act as multitaskers, with the same EV population carrying out niche formation and EMT induction simultaneously. Integrins α6β4/α6β1 and CD44, two molecules on the surface of EVs, can function as postal codes that direct EVs specifically to the lungs (Altei *et al*., 2020; Hoshino *et al*., 2015; Wang *et al*., 2024a; Kesharwani *et al*., 2021; Johnson *et al*., 2018), while these EVs simultaneously carry molecules that support niche development and EMT. For example, *via* stimulating the TLR4–NF‐κB–IL‐6/CCL2 axis, CAV1‐rich EVs not only improve uptake by lung epithelial cells but also concurrently promote EMT and niche formation (Lin *et al*., 2024a).

Nonetheless, evidence also indicates that the cargo composition of EVs may differ according to cellular state and microenvironment. For example, exosomes containing specificity protein 1 (SP1) generated by chronic stress are absorbed by pulmonary neutrophils, thereby remodelling the microenvironment through the activation of IL‐1β secretion *via* the TLR4–NF‐κB pathway (Zhang *et al*., 2025), perhaps involving several EV subpopulations working in concert. Clarifying the molecular underpinnings of BC metastasis thus requires an understanding of the several EV subpopulations engaged in EMT induction and niche creation, as well as how they interact. Additionally, the ‘multitaskers’ hypothesis faces stoichiometric challenges given the limited intra‐vesicular volume available to harbour sufficient concentrations of diverse cargo simultaneously. This physical constraint suggests that a ‘synergistic subpopulations’ hypothesis may be more plausible than a model of the ‘single super‐vesicle’. The low payload capacity of small EVs casts doubt on the idea that a single EV may both promote EMT and stimulate the microenvironment. The secretion of several EV subpopulations, with some devoted to long‐distance microenvironmental regulation and others to autocrine/paracrine EMT induction, may provide a more plausible biological explanation. A crucial next step thus will be to distinguish between multitasking vesicles and synergistic subpopulations.

### (2) Temporal dynamics: the EV ‘first wave’ and the CTC ‘second wave’

Tumour metastasis represents a highly orchestrated and dynamic process that involves meticulous coordination across various phases. Recent studies suggest that the onset of tumour metastasis is not a stochastic event, but rather a process in which EVs secreted by tumour cells at the primary site function as a ‘first wave’. These EVs remotely modify the microenvironment of the target organ, establishing a pre‐metastatic niche, while circulating tumour cells (CTCs) that have undergone EMT and shedding function as the ‘second wave’, effectively colonising this altered environment (Entenberg *et al*., 2023; Gerstberger *et al*., 2023; McGinnis *et al*., 2024; Xiao *et al*., 2025; Fares *et al*., 2020; Bonin *et al*., 2022; Li *et al*., 2023; Yousefi *et al*., 2021).

Primary cancer cells secrete EVs (exosomes, microvesicles, etc.) that have distant impacts on the pulmonary microenvironment. CAV1, Wnt7a, ephrin type‐A receptor 2 (EPHA2), and miR‐18b are among the particular molecular signals carried by these EVs that influence the polarisation of pulmonary immune cells (such as neutrophils and macrophages), encourage angiogenesis, and alter the extracellular matrix. All of these factors work together to create a pre‐metastatic environment that is favourable for cancer cell colonisation (Lin *et al*., 2024a; Zhang *et al*., 2025; Jiang *et al*., 2024; Yofe *et al*., 2023; Han *et al*., 2022; Adnani *et al*., 2022; Li *et al*., 2021). For example, CAV1 derived from BC facilitates lung metastasis by modulating integrin α6β4 and recruiting polarised tumour‐associated neutrophils; SP1 (+) exosomes induced by chronic stress from tumours polarise IL‐1β (+) neutrophils, thereby enhancing BC lung metastasis; exosomal miR‐18b from CAFs promotes BC invasion and metastasis through regulation of TCEAL7; additionally, exosomal EPHA2, originating from highly metastatic BC cells, activates the AMP‐activated protein kinase (AMPK) pathway *via* Ephrin A1–EPHA2 positive feedback signalling to promote angiogenesis (Lin *et al*., 2024a; Zhang *et al*., 2025; Yan *et al*., 2021; Han *et al*., 2022). These EVs are released during the initial phases of tumour metastasis, facilitating subsequent tumour cell colonisation.

Following EMT, tumour cells detach from the primary tumour, enter the bloodstream, and develop into CTCs. EMT is a crucial stage for tumour cell detachment from the original site and entry into the circulation because it gives tumour cells the ability to invade and migrate while also causing them to shed epithelial traits and develop a mesenchymal phenotype (Li *et al*., 2025; Xie *et al*., 2023; Raymond *et al*., 2025; Fares *et al*., 2020; Bonin *et al*., 2022). In order to implant these CTCs effectively, the pulmonary microenvironment must be habitable and remodelled by EVs. For example, in BC metastasis, pulmonary fibroblasts operate as ‘soil fertilisers’, whereas EVs can alter pulmonary fibroblast activity to promote CTC implantation (Houthuijzen & de Visser, 2022; Xiao *et al*., 2025). Research suggests that lung‐specific metastasis entails co‐evolution between tumour cells and the pulmonary microenvironment, wherein EV‐mediated remodelling of the microenvironment establishes a dynamic balance with the colonisation potential of CTCs (Xiao *et al*., 2025). During BC metastasis, *in vivo* imaging studies show that lung immune remodelling proceeds in a time‐specific manner: EV‐mediated microenvironmental remodelling begins first, followed by effective CTC colonisation (McGinnis *et al*., 2024).

The precise regulatory mechanisms of cancer metastasis are revealed by the spatiotemporal dynamic process of the ‘first wave’ of EVs and the ‘second wave’ of CTCs. The pulmonary milieu must be modified for CTC implantation to be successful, and the ability of CTCs to implant depends on whether they undergo EMT and develop the necessary migratory and implantation skills. Accumulating evidence indicates that triggering receptor expressed on myeloid cells 2 (TREM2)‐positive macrophages are crucial in controlling the boundaries of lung metastases in BC, and macrophage‐mediated aromatase activity encourages the development of pre‐metastatic microenvironments, further supporting the close connection between microenvironment remodelling and CTC engraftment (Jiang *et al*., 2024; Yofe *et al*., 2023). This dynamic continuum provides new approaches to metastasis prevention and therapy in addition to explaining why some cancer cells spread to specific organs (organ‐specific metastasis). For example, CTC engraftment may be hindered by preventing EV‐mediated pre‐metastatic niche development (Shang *et al*., 2025; Steinbichler *et al*., 2019).

### (3) Evolutionary implications: how the EV–EMT–niche axis drives metastatic fitness

The EV–EMT–niche axis is a dynamic, selective evolutionary circuit from the standpoint of tumour evolution. It permits the survival of the fittest among cancer cells during metastasis by methodically identifying and amplifying subclones with the ability to spread. In addition to explaining the organ‐specific selection of metastases, this demonstrates how cancer cells use intercellular communication to evolve adaptively.

EVs are essential mediators in the tumour microenvironment that benefit cancer cells by carrying a variety of molecules. TEVs provide a pre‐metastatic niche that is suitable for particular subclonal colonisation within the original tumour by not only inducing cancer cells to undergo EMT for migratory capability but also by deploying EMT‐associated molecules to distant organs as ‘advance guards’. Thus, EMT gives cancer cells the ability to migrate, and EV‐mediated microenvironmental remodelling selects for subclones that are most suitable for colonising particular organs. Together, these two processes create a positive feedback loop. Through intercellular communication facilitated by EVs, cancer cells actively design and adjust the metastatic microenvironment.

For the EV–EMT–niche axis, tumour heterogeneity offers abundant evolutionary material. The varied metastatic potential of different subclones is determined by the specificity with which EVs generated by various BC subtypes induce EMT. EVs derived from TNBC cells, for example, include certain miRNAs (such as miR‐106a–5p) that can specifically increase EMT and metastatic potential in specific subclones (Ansieau, 2013). Simultaneously, the targeting precision of these EVs to remote organs is affected by their molecular composition, allowing subclones with distinct EV profiles selectively to colonise specific organs (Shen & TanTai, 2024; Yang *et al*., 2017).

The ability of the EV–EMT–niche axis to customise microenvironments thus provides an evolutionary advantage for the colonisation of particular subclones. For example, pulmonary epithelial cells can be reprogrammed by miR‐451a transported by EVs, resulting in lung fibrosis and a permissive pathological environment for the colonisation of cancer cells (Jeong *et al*., 2022).

It is noteworthy that the considerable heterogeneity of EVs, which arise from a variety of sources, range widely in size, and have extremely variable compositions, offers abundant molecular diversity for the evolution of tumours. This makes it possible for the EV–EMT–niche axis to identify and select for subclones with particular metastatic potential. However, technical limitations currently make it difficult to elucidate precisely the involvement of particular EV subpopulations in tumour evolution. These limitations include difficulties in EV extraction, characterisation, and *in vivo* tracking (Zheng *et al*., 2024; Wang *et al*., 2024b; Robinson *et al*., 2023; Franco *et al*., 2023; Skoczylas *et al*., 2024; Welsh *et al*., 2024; Kang *et al*., 2025; Willms *et al*., 2018).

Future studies should concentrate on clarifying the dynamic mechanisms of EVs in the evolution of tumours, especially by utilising next‐generation technologies (like spatial ‐omics, single‐cell analysis, and single‐vesicle analysis) to understand EV heterogeneity and its functional role in the selection of metastatic subclones (Fathi *et al*., 2023; Wang *et al*., 2025; Welsh *et al*., 2023; Sharma *et al*., 2020; Ridolfi *et al*., 2020; Kowkabany & Bao, 2024; He *et al*., 2019; Penders *et al*., 2018, 2021). In addition to improving our knowledge of the biology of cancer metastasis, a deeper comprehension of the evolutionary mechanisms within the EV–EMT–niche axis will offer a theoretical basis for creating innovative therapeutic approaches that target the metastatic process. According to this integrated viewpoint, tumour metastasis is a dynamic evolutionary process in which EVs play a crucial role as communication mediators that promote the adaptive evolution of tumour cells within the metastatic microenvironment.

## V. CHALLENGES AND FUTURE PERSPECTIVES: UNVEILING NEW FRONTIERS IN EV BIOLOGY

### (1) Overcoming methodological hurdles: from bulk analysis to single‐vesicle resolution

**Fig. 3:**
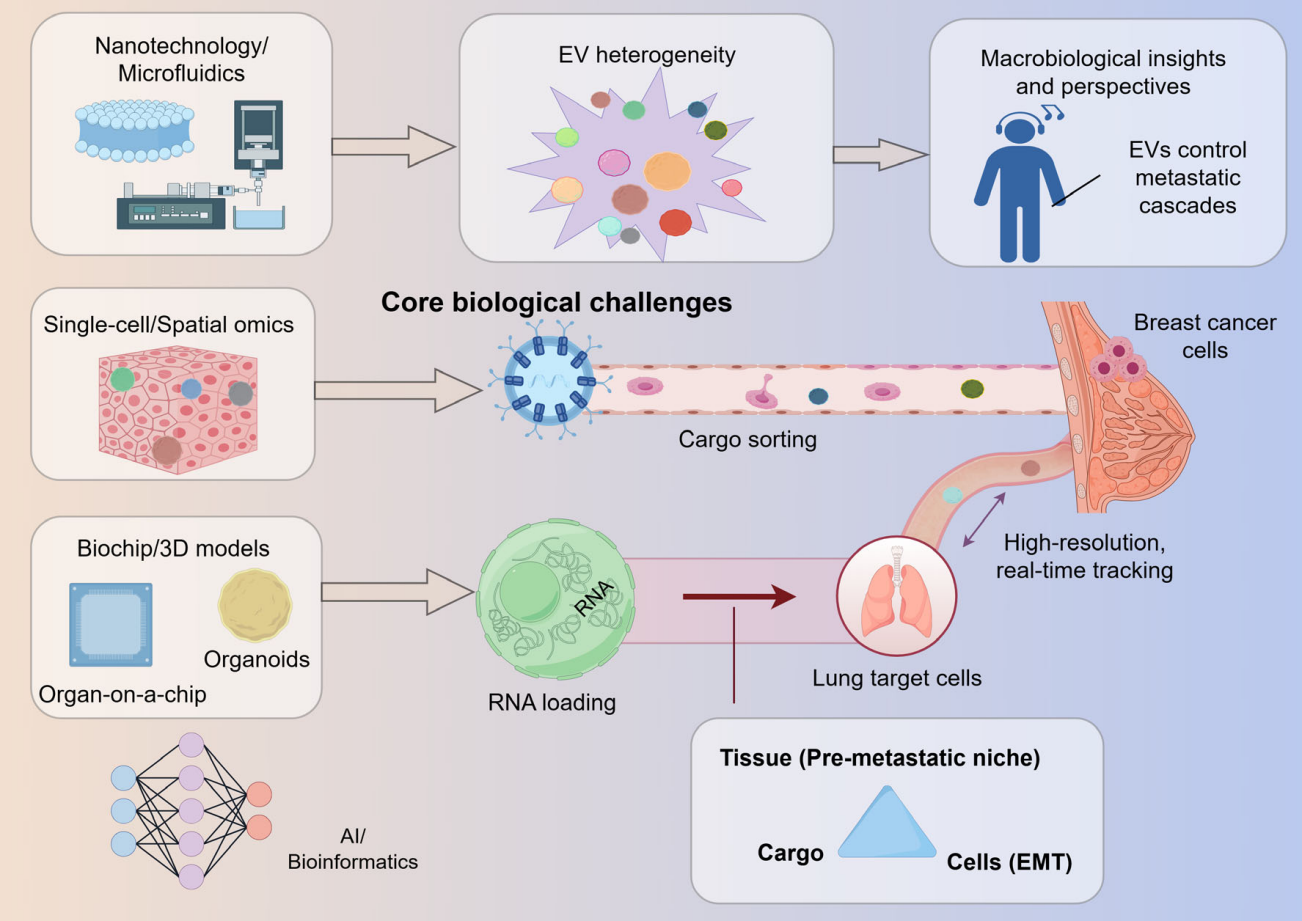
Depiction of contemporary technologies employed to examine the biological processes of extracellular vesicles in breast cancer metastasis. Biochips and 3D organoid models, single‐cell and spatial ‐omics, nanotechnology and microfluidics, as well as AI and bioinformatics, are all illustrated in the left panel. The central section illustrates the core biological challenges, including the high‐resolution dynamic tracking of vesicles released by breast cancer cells circulating to target cells in the lungs, an enlarged view of cargo‐sorting mechanisms (such as RNA loading), and extracellular vesicle heterogeneity. The macrobiological insights into the control of metastatic cascades by extracellular vesicles are illustrated in the right panels. These insights emphasize multi‐level regulatory networks that involve cargo molecules, cellular epithelial–mesenchymal transition, and tissue‐level pre‐metastatic niche formation. Abbreviations: AI, artificial intelligence; BC, breast cancer; EMT, epithelial–mesenchymal transition; EVs, extracellular vesicles.

The lack of consistent separation and characterisation techniques is a major obstacle to current EV research. EV purity and recovery rates are severely limited by current methods like size‐exclusion chromatography and ultracentrifugation, raising questions about the reproducibility of study results (Simon *et al*., 2020b). The development of nanopore and microfluidic sensing technologies provides new ways to overcome this bottleneck. These new approaches have the potential to allow for high‐throughput, high‐purity EV isolation and identification (Fig. 3). The current limitations not only obscure the specific role of lipid cargoes but also impact the broader challenge of defining functional EV subpopulations.

Technological developments in *in vivo* tracking will be essential to understanding the dynamic behaviour of EVs in the tumour microenvironment. Currently, high‐resolution live‐imaging methods are still in their infancy and cannot track EV transit, targeting, or biodistribution in real time. To understand accurately the spatiotemporal dynamics of EVs within the BC metastatic cascade, future developments will need to include more sensitive fluorescent labelling techniques and imaging technology.

Since EVs are complex biological signal carriers due to their multifaceted molecular composition, including proteins, nucleic acids, lipids, and metabolites, research approaches that can resolve many cargo molecules simultaneously are required. To comprehend EV‐mediated EMT regulatory networks, for example, accurate detection of miRNAs like miR‐21, miR‐23b, and miR‐106a‐5p will be essential (Ma *et al*., 2010; Akinsipe *et al*., 2024; Samuels *et al*., 2023). More sophisticated analytical techniques are also needed to understand the regulatory functions of lipid components carried by EVs, including glycerophospholipids, sphingolipids, and cholesterol (Shang *et al*., 2025).

In order to clarify EV heterogeneity and its precise regulatory mechanisms in BC metastasis, future research should focus on developing single‐vesicle analytic tools. BC metastasis research will enter a new phase of precision if we can combine single‐vesicle analysis with *in vivo* tracking technologies, which will offer unprecedented insights into EV‐mediated intercellular communication pathways.

### (2) Decoding complexity: new models and computational integration

The incorporation of novel models and computational biology has demonstrated that EVs modulate intricate EMT networks within the tumour microenvironment *via* various molecular mechanisms (Ma *et al*., 2010; Akinsipe *et al*., 2024; Samuels *et al*., 2023; Ma *et al*., 2024), offering critical insights into the biological underpinnings of BC metastasis. The dynamic role of EVs in the tumour microenvironment calls for models that are more physiologically accurate in order to investigate how they work in intricate tissue contexts. For example, the pre‐metastatic lung microenvironment for BC is established by the reprogramming of diverse lung stromal cells and immune cells through complex molecular interactions within the dynamic signalling networks controlled by EVs (Becker *et al*., 2016; Ye *et al*., 2023; Liu *et al*., 2021b).

The majority of current research uses two‐dimensional cell culture techniques, which do not accurately represent the dynamic roles that EVs play inside the tumour microenvironment *in vivo*. The common use of EVs at supraphysiological doses in *in vitro* research presents another challenge for the interpretation of mechanisms. The involvement of EVs in processes like EMT may be overstated due to the possibility that large doses will activate cellular uptake mechanisms that are dormant under physiological conditions. Thus, creating models that more closely resemble physiological settings – like organ‐on‐a‐chip systems and three‐dimensional organoids – will help clarify the dynamic roles of EVs in the tumour microenvironment and more accurately model interactions between BC cells and the pulmonary microenvironment.

Simultaneously, combining artificial intelligence algorithms with multi‐omics data (such as spatial transcriptomics, proteomics, and metabolomics) could help to uncover important molecular patterns linked to EV activities and their role in metastasis. Use of computational biology techniques to analyse these complex data could uncover connections between particular miRNA and protein combinations inside EVs and metastatic potential, providing new methods for the accurate prediction and individualised therapy of BC metastasis.

### (3) A new biological paradigm: EVs as central nodes in cancer ecology and evolution

Research on EVs has advanced beyond their basic function as biomolecular carriers, and EVs now represent a crucial lens for elucidating the fundamental mechanisms by which tumours attain internal coordination and modify their external environment. Dynamic communication through EVs among cells within the tumour microenvironment not only orchestrates the synergistic interactions of heterogeneous cellular populations within the tumour but also modifies the ecological relationships between the tumour microenvironment and distal organs. This offers comprehensive support for tumour progression and organ‐specific metastasis.

We now know that EVs secreted by various tumour microenvironment cells control the EMT process by the transfer of proteins, non‐coding RNAs, and other compounds. Nevertheless, the precise biochemical pathways and unique EV subpopulations that cause these effects are still not well understood (Jeppesen *et al*., 2023). This knowledge gap significantly limits our ability to understand the mechanistic role of EVs in the ecology and evolution of tumours. Reassessing the functions of EVs in the tumour system from a ‘superorganism’ perspective is necessary to progress beyond this obstacle.

In the future, fundamental research exploring EVs could greatly expand our cognitive boundaries in cancer biology and life sciences as technical obstacles are removed and interdisciplinary collaboration grows. In addition to revealing the internal coordination and external environmental remodelling principles of tumours as ‘superorganisms’, a thorough understanding of the ecology and evolutionary mechanisms of EV‐mediated tumours will open up previously unexplored scientific frontiers by offering fresh insights into the intricate information exchange and systemic evolution that occur within living systems.

## VI. CONCLUSIONS

(1) The complexity of BC lung metastasis lies in the intricate communication network between tumour cells and remote organs, with EVs acting as key mediators within this system. This review systematically elucidates the mechanisms by which EVs functionally connect cellular intrinsic plasticity with adaptive remodelling of remote organ niches, thereby orchestrating the cascade from primary tumour to pulmonary metastasis. As highly effective molecular mediators, TEVs not only initiate the EMT programme by transporting essential molecules such as TGF‐β, Wnt signalling proteins, and miR‐9, but also enhance the migratory and invasive potential of cancer cells. They also serve as a vanguard, reaching the lungs ahead of the tumour cells to educate stromal cells and establish a pre‐metastatic niche that facilitates metastatic colonisation and growth.
(2) Increased comprehension of the EV–EMT–niche axis will elucidate essential principles of BC metastasis and offers a new framework for investigating intercellular communication, organogenesis, and tissue equilibrium. The intricate molecular distribution facilitated by EVs (proteins, non‐coding RNAs, lipids, and metabolites) illustrates the sophistication and accuracy of intercellular communication, revealing new mechanisms that regulate the tumour microenvironment. The tumour microenvironment is a dynamic, bidirectional, and synergistic regulatory network. In this context, EVs function as the primary intermediaries, facilitating accurate control from the molecular to the tissue level.
(3) Future studies should focus on EV heterogeneity, *in vivo* dynamic mechanisms, and their functions in the evolution of tumours. Significantly, the advancement of single‐EV analytical methods will offer hitherto unavailable accuracy in understanding EV‐mediated metastatic regulation. These methods will expand our knowledge of metastasis as a biological process and offer a theoretical basis for creating intervention techniques based on EVs, potentially opening up new possibilities for BC patients to receive precise treatments. In addition to changing how we think about BC metastasis, the function of EVs as orchestrators provides important new information about intercellular communication in biological systems.

## AUTHOR CONTRIBUTIONS

J.L. and R.S.N. contributed equally as first authors. Conceptualisation: J.L. and G.G.; Writing – Original Draft: J.L., R.S.N., J.S., C.F., D.D.V., P.H., H.L., H.Y., C.P., X.W., X.G. and X.X.; Writing – Review and Editing: J.L. and G.G.; Visualisation: J.L. and J.S. All authors read and approved the final version.

## Data Availability

Data sharing not applicable to this article as no datasets were generated or analysed during the current study.
